# Supplementary material for: Structural insights into actin filament recognition by commonly used cellular actin markers
Source: EMBO J. 2020 Jun 22;39(14):e104006. doi: 10.15252/embj.2019104006 (PMC7360965; doi:10.15252/embj.2019104006)
Supplement: Supplementary file 1 — Appendix [file EMBJ-39-e104006-s001.pdf]

Index of Appendix Figures

1. Appendix Figure S1: F-actin-ADP apo versus phalloidin bound maps
2. Appendix Figure S2: LifeAct versus actin nucleotide state titration and mutation analysis
3. Appendix Figure S3: Co-sedimentation gels of UTRN mutations.
4. Appendix Figure S4: Utrophin structural comparison

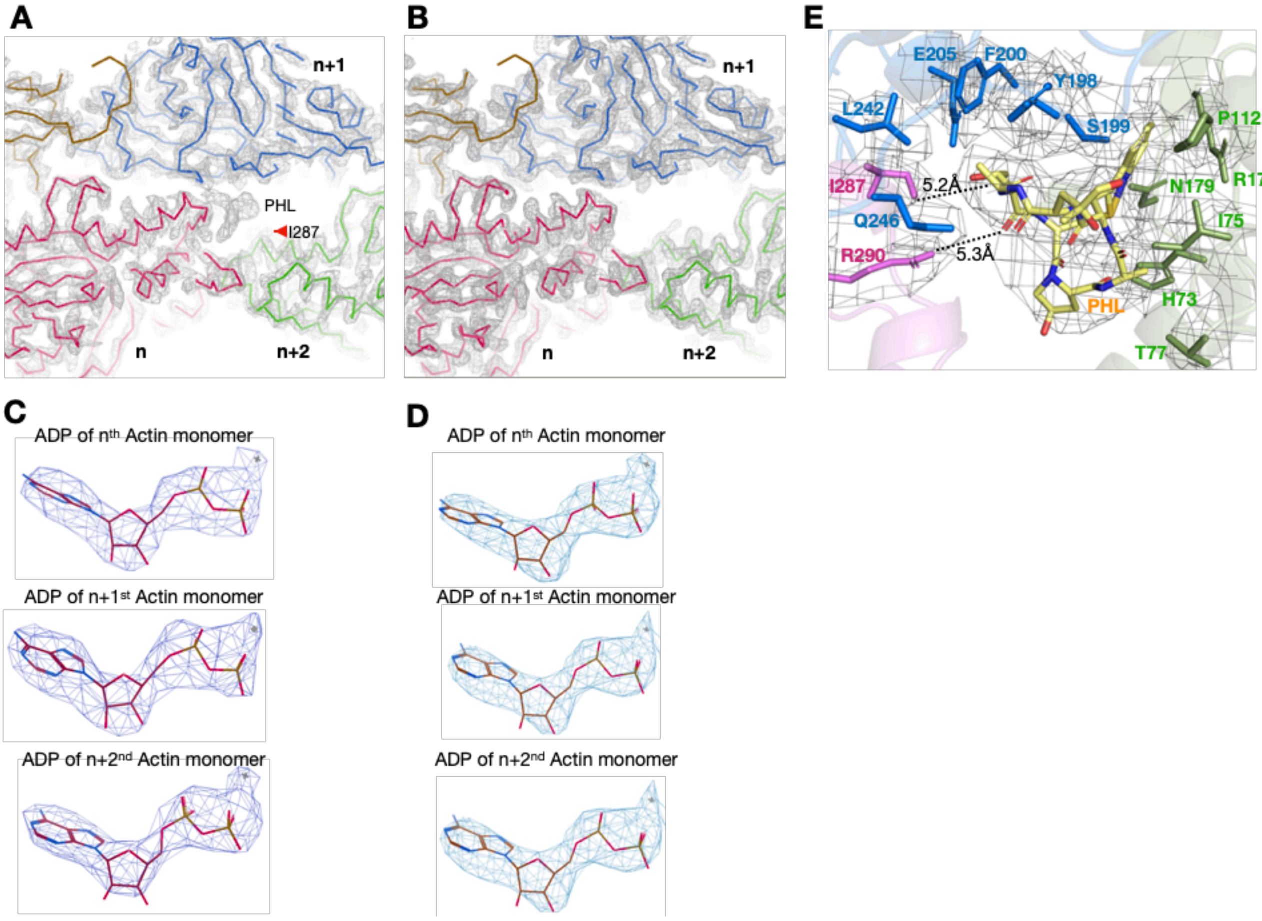

Appendix Figure S1: F-actin-ADP apo versus phalloidin bound maps. A. and **B.** F-actin ADP apo versus phalloidin maps. The density of phalloidin is indicated with PHL and the I287 residue is marked as red arrow. **C** and **D**. The density for ADP in the structures of actin monomers in apo and phalloidin maps respectively. **E.** Phalloidin binding pocket with map as shown in Figure 1B and 1C, maps were made in Pymol contoured at σ 5.0.

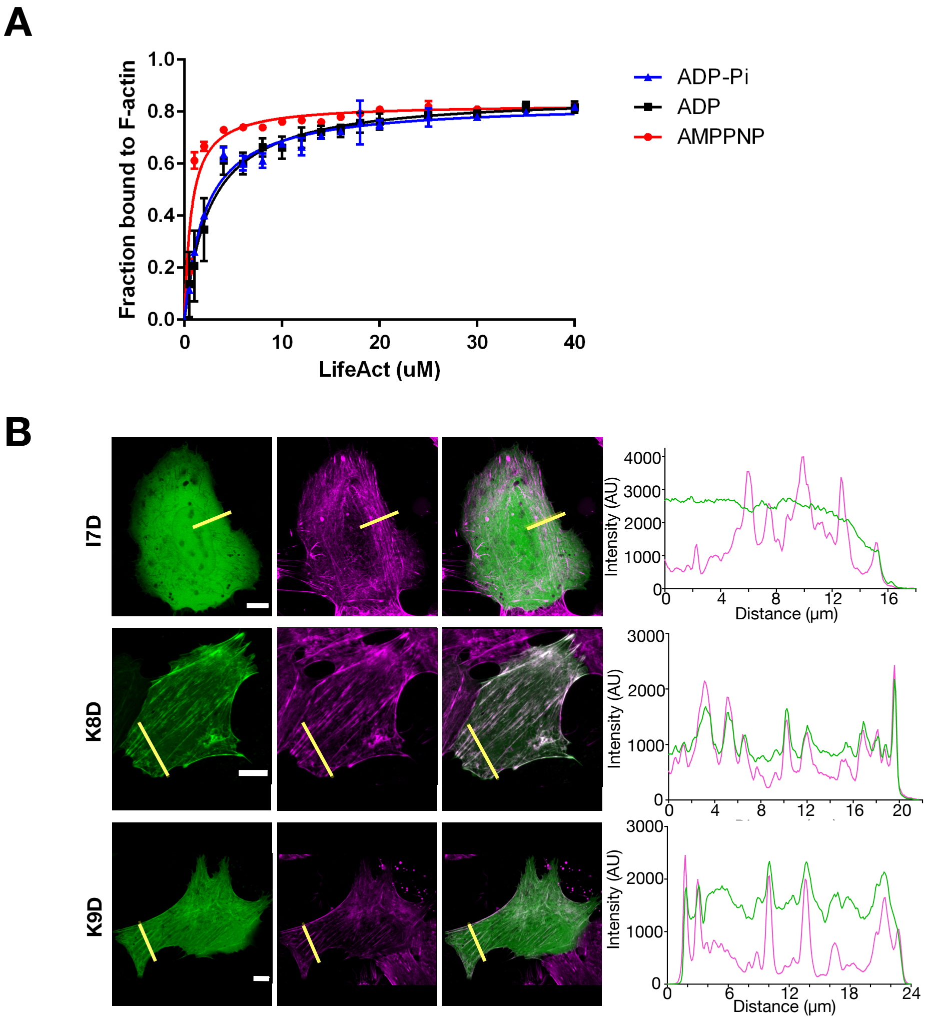

Appendix Figure S2: LifeAct versus actin nucleotide state titration and mutation analysis. A. Binding affinities of LifeAct towards Actin-ADP (2.4µM), ADP-Pi (2.1µM), and AMPPNP (0.8µM), calculated from titration data of co-sedimentation assays. *Note: The discrepancy in AMPPNP affinity might be due to the variations in actin polymerization with AMPPNP, thus leading to increased F-actin and bound lifeAct in the titration assay.* **B.** Confocal images of U2OS cells transiently expressing lifeAct-GFP wild type and mutants of lifeAct residues interacting with F-actin, cells were additionally stained with SiR-actin to confirm the actin filaments. The line scan as indicated with yellow line on the cells shows the extent of lifeAct (green) and SiR-actin (magenta) co-staining of actin structures. Scale bar = 5µm.

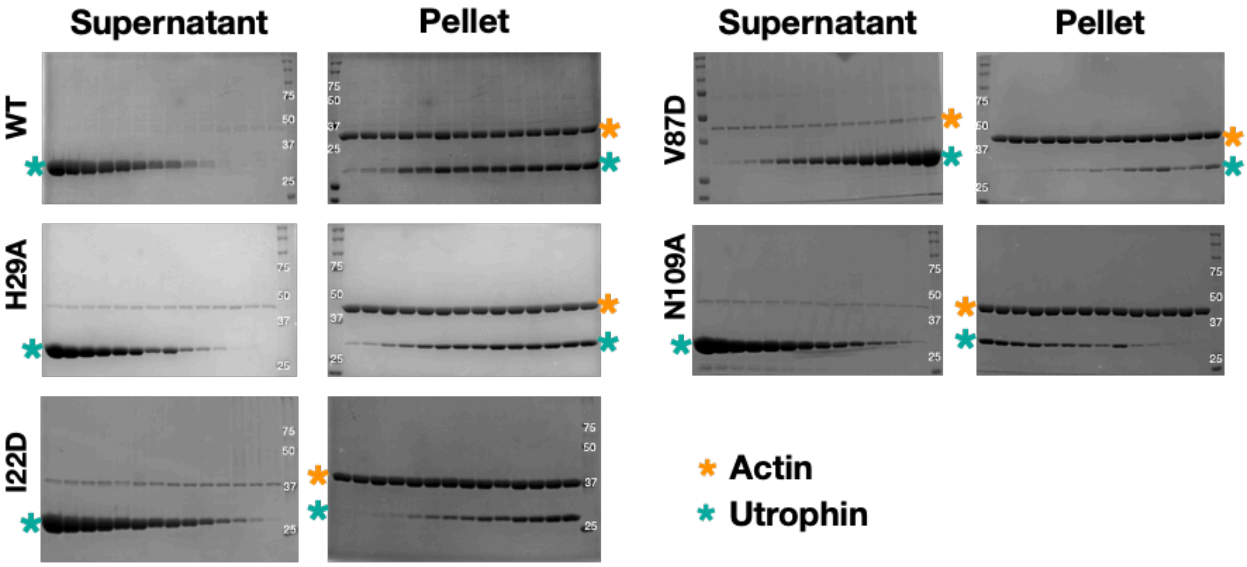

Appendix Figure S3: Co-sedimentation gels of UTRN mutations. Uncropped gel images of co-sedimentation experiments with F-actin versus utrophin wildtype and mutants used in calculating the K_d shown in Figure 5B.

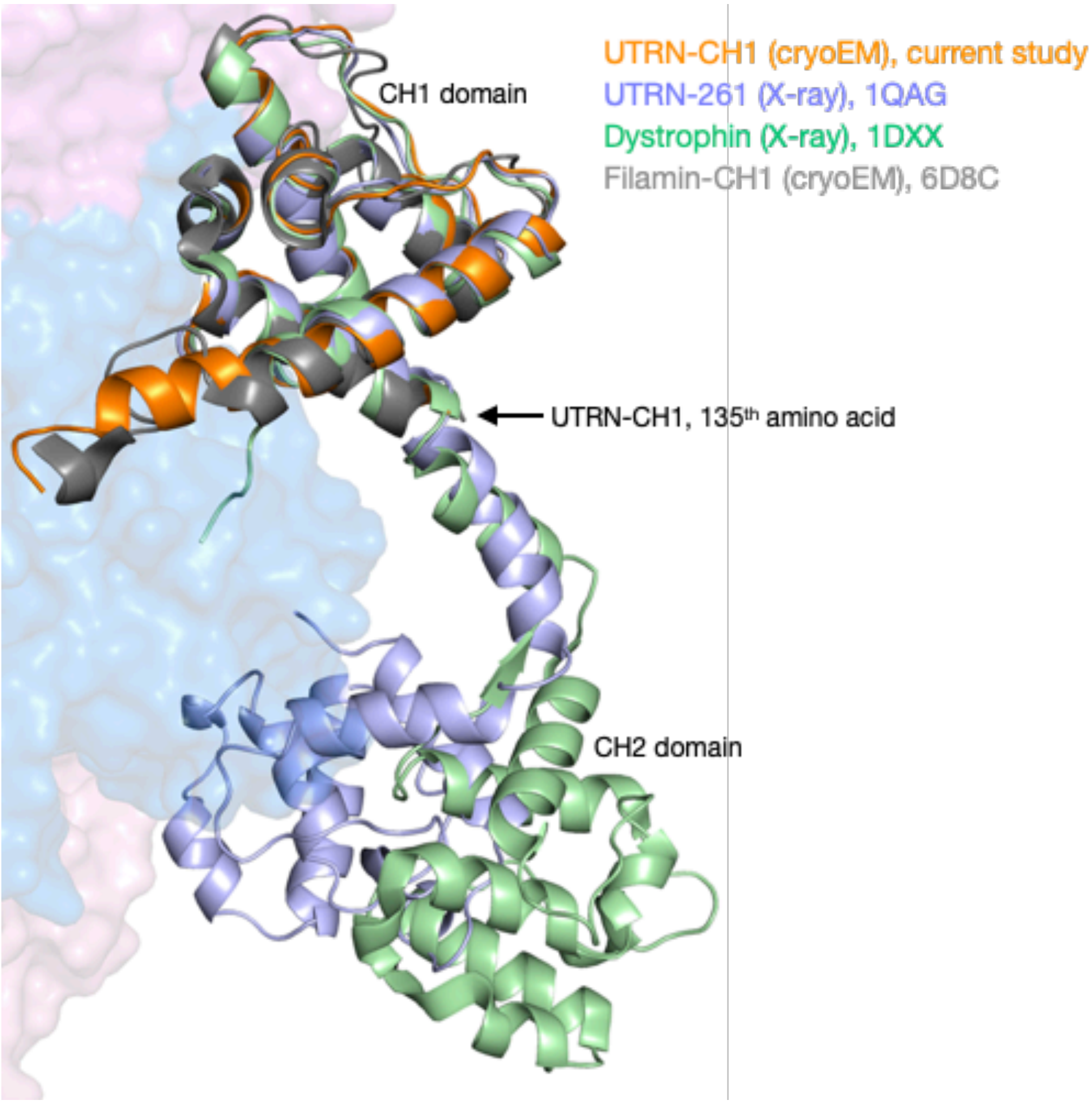

Appendix Figure S 4: Utrophin structural comparison. Utrophin-CH1 cryoEM structure comparison with UTRN-261 and dystrophin X-ray structures and filamin-CH1. The CH1 of all the structures overlay with <1Å rmsd deviation. The CH2 domain of UTRN- X-ray structure will sterically interfere with F-actin in its current conformation.
