# Supplementary material for: Structural insights into actin filament recognition by commonly used cellular actin markers
Source: EMBO J. 2020 Jun 22;39(14):e104006. doi: 10.15252/embj.2019104006 (PMC7360965; doi:10.15252/embj.2019104006)
Supplement: Supplementary file 2 — Expanded View Figures PDF [file EMBJ-39-e104006-s002.pdf]

Expanded View Figures

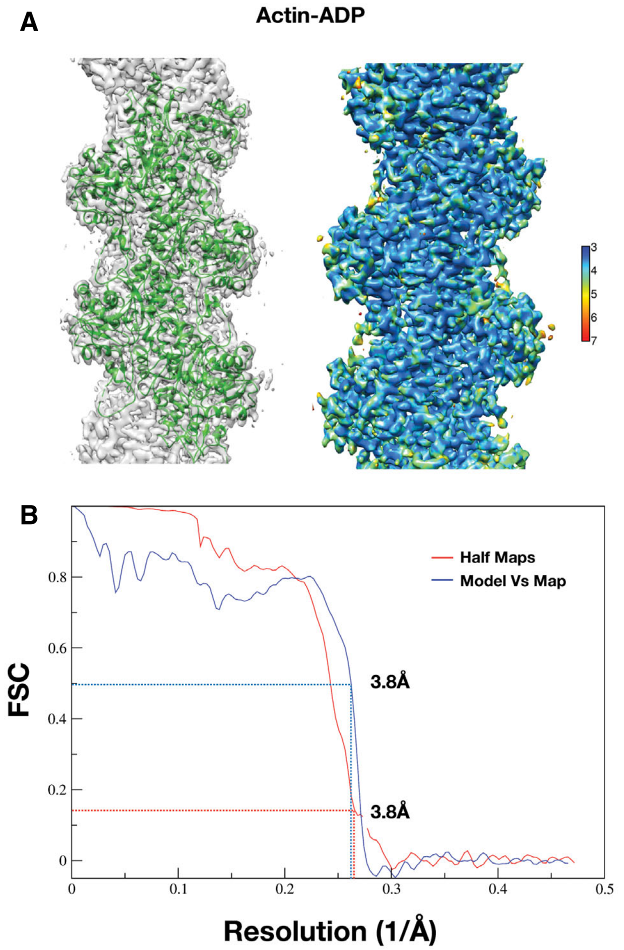

Figure EV1. F-actin apo and phalloidin-bound structure validation and quality control.

A–D (A, C) The overlay of the model (green) on the cryoEM map in gray and local resolution map of actin:ADP (apo) and actin:ADP-phalloidin structures, respectively. The red density is the difference density of phalloidin in panel (C). (B, D) FSC plots of the half-maps and the map versus model for the Actin–ADP and Actin–ADP–phalloidin, respectively. The resolutions were estimated based on 0.143 criteria for the half-maps and 0.5 for the model versus map.

 © 2020 The Authors

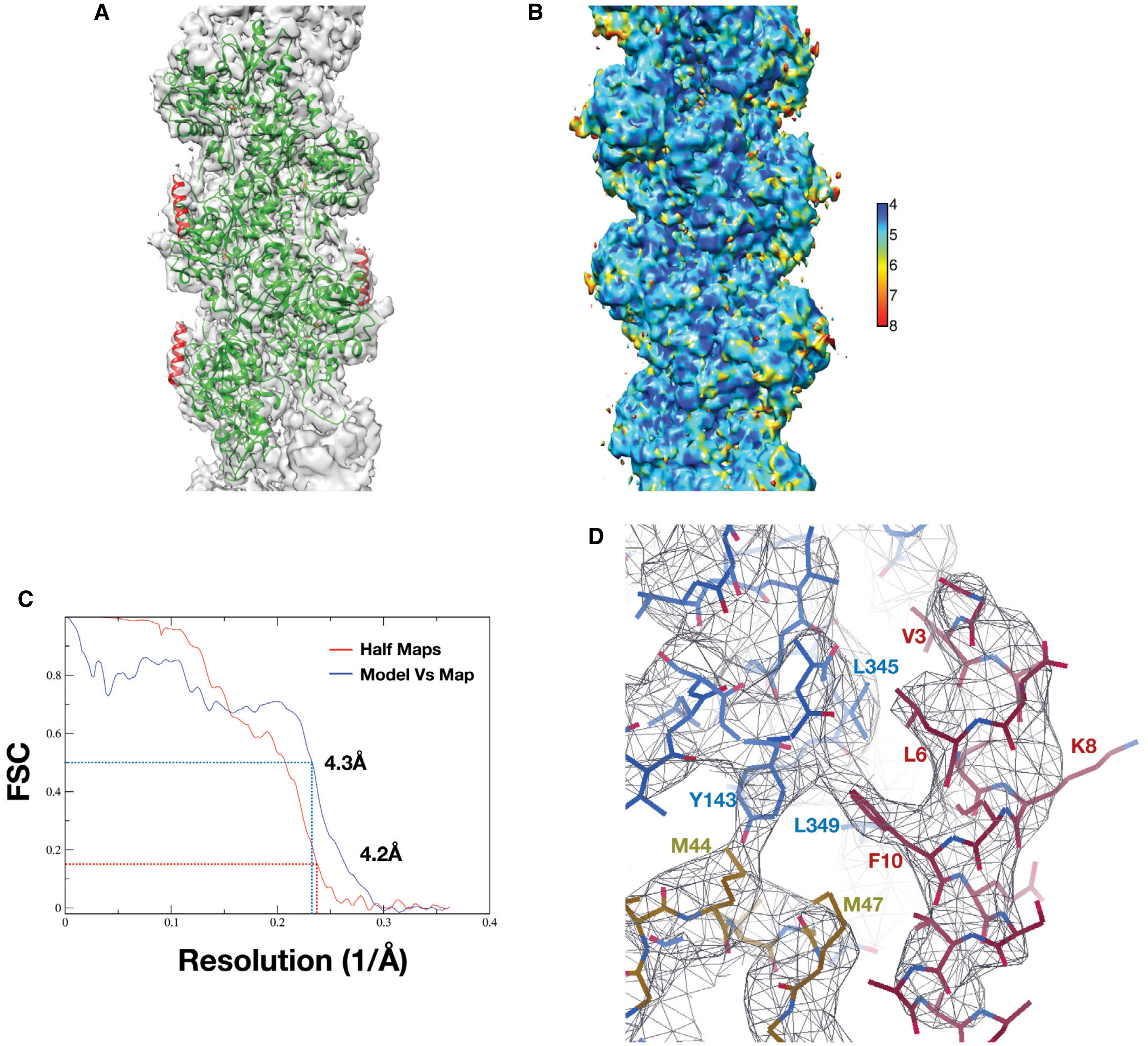

Figure EV2. F-actin:lifeAct complex maps, structure validation and quality control.

A Overlay of model with map, actin, and lifeAct are colored as green and red, respectively.

B Local resolution of lifeAct-bound F-actin map with color gradient chart determined with Resmap.

C FSC plots of the half-maps (0.143) and the map versus model (0.5) for F-actin:lifeAct structure.

D Closer view of lifeAct:F-actin interaction with map, key residues marked.

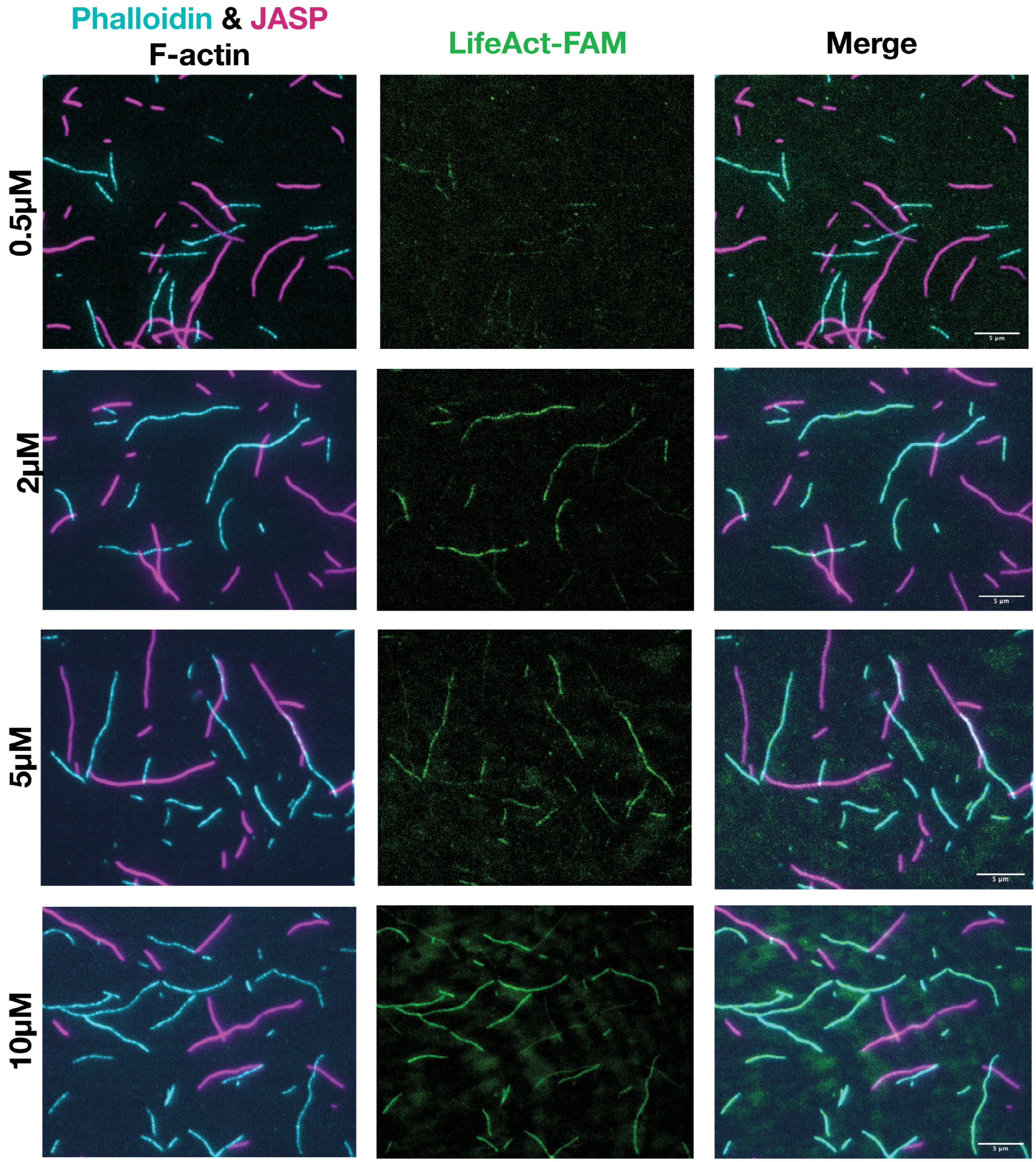

Figure EV3. TIRF images of lifeAct bound to F-actin with phalloidin and jasplakinolide.

Representative TIRF images of lifeAct- versus phalloidin- and jasplakinolide-bound F-actin for concentrations as indicated. Scale bar = 5 µm.

 © 2020 The Authors

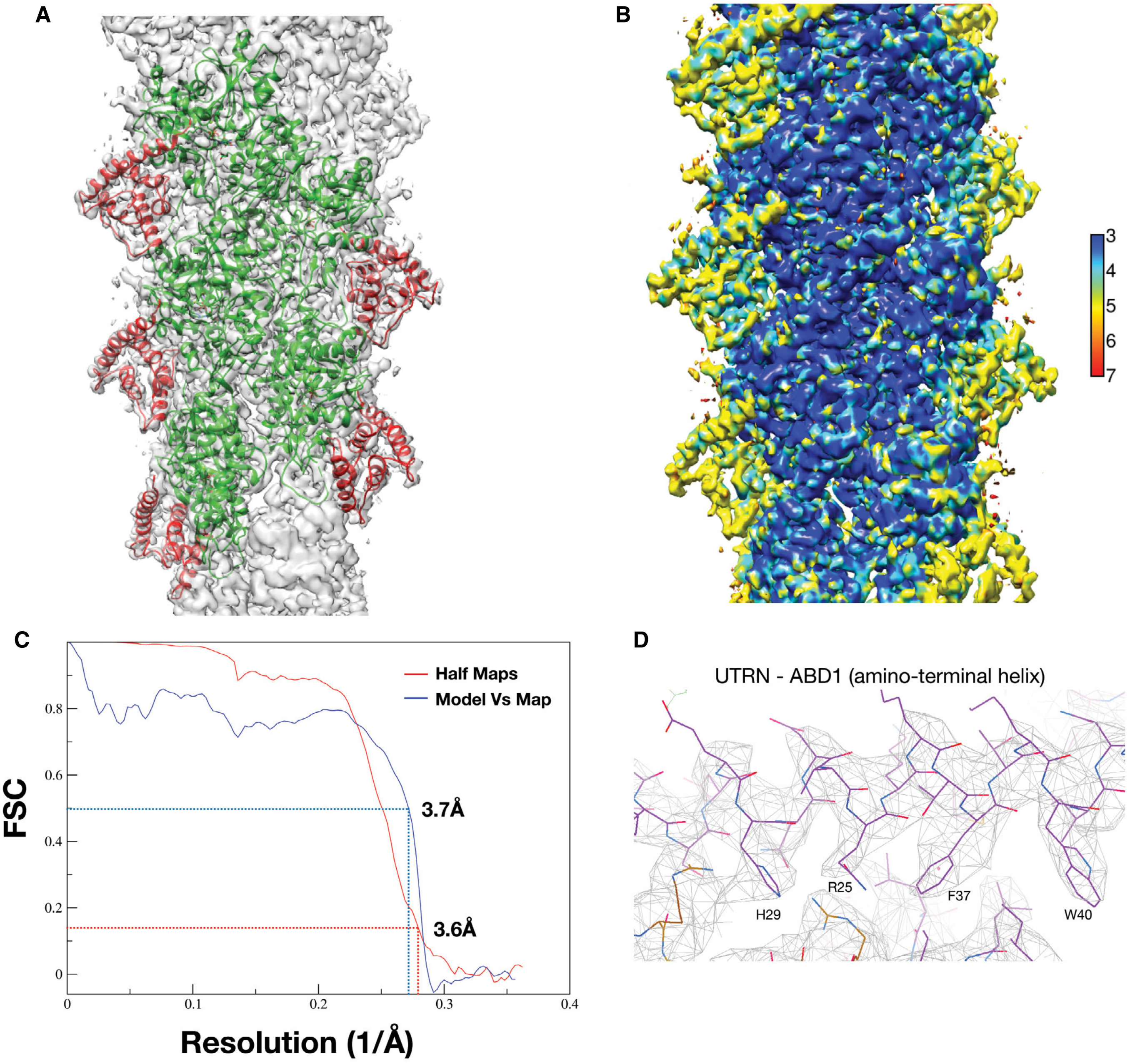

Figure EV4. Utrophin:F-actin complex maps, structure validation, and quality control.

A Overlay of model with map, actin and utrophin CH1 are colored as green and red, respectively.
B Local resolution of utrophin CH1-bound F-actin map with color gradient chart as indicated.
C FSC plots of the half-maps (0.143) and the map versus model (0.5) for F-actin:utrophin CH1 structure.
D Closer view of ABD1 (amino-terminal helix) utrophin map with key residues marked.

© 2020 The Authors
